# Role of Jagged1-mediated Notch Signaling Activation in the Differentiation and Stratification of the Human Limbal Epithelium

**DOI:** 10.3390/cells9091945

**Published:** 2020-08-22

**Authors:** Sheyla González, Maximilian Halabi, David Ju, Matthew Tsai, Sophie X. Deng

**Affiliations:** Cornea Division, Stein Eye Institute, University of California, Los Angeles, CA 90095, USA; s.gonzalez@jsei.ucla.edu (S.G.); maxhalabi32@gmail.com (M.H.); davis4717@gmail.com (D.J.); thew8888@gmail.com (M.T.)

**Keywords:** Notch signaling, stratification, differentiation, limbal epithelium, limbal stem cells

## Abstract

The Notch signaling pathway plays a key role in proliferation and differentiation. We investigated the effect of Jagged 1 (Jag1)-mediated Notch signaling activation in the human limbal stem/progenitor cell (LSC) population and the stratification of the limbal epithelium in vitro. After Notch signaling activation, there was a reduction in the amount of the stem/progenitor cell population, epithelial stratification, and expression of proliferation markers. There was also an increase of the corneal epithelial differentiation. In the presence of Jag1, asymmetric divisions were decreased, and the expression pattern of the polarity protein Par3, normally present at the apical-lateral membrane of basal cells, was dispersed in the cells. We propose a mechanism in which Notch activation by Jag1 decreases p63 expression at the basal layer, which in turn reduces stratification by decreasing the number of asymmetric divisions and increases differentiation.

## 1. Introduction

The corneal epithelium is the outermost layer of the eye that functions as a barrier to prevent infections and maintains the corneal transparency. The corneal epithelium is regenerated by a population of stem cells, the limbal epithelial stem/progenitor cells (LSCs), which reside at the sclerocorneal junction between the conjunctiva and cornea called limbus [1,2]. LSCs maintain the corneal homeostasis throughout life. Asymmetric divisions of the LSCs produce a stem cell that remains at the basal layer and a more differentiated daughter cell at a suprabasal level. The suprabasal cells undergo multiple rounds of division and progressively lose stemness to terminally differentiate towards the surface of the epithelium and centripetally to replenish the corneal epithelium [3,4,5]. This highly regulated corneal epithelial cell differentiation process and the underlying signaling mechanisms are still poorly understood, albeit an extensive body of literature has been published. The understanding of this tight regulation of the corneal epithelium would also help developing new treatments with a better outcome by modulating specific signaling pathways in vitro. The mechanisms underlying the regulation of differentiation in other epithelia like the epidermis have been well studied [6].

Notch signaling is a developmentally conserved signaling pathway that controls multiple cell processes such as proliferation, differentiation, cell fate and cell death. Although Notch signaling has a very simple design in terms of the number of proteins involved in the pathway, it is a very complex and versatile pathway based on the different cell responses it can trigger [7,8]. Activation of Notch signaling by binding of the ligand to the Notch receptors triggers the cleavage of the Notch receptor by γ-secretase. The cleaved intracellular domain of the Notch receptor subsequently translocates into the nucleus and promotes the transcription of Notch-dependent target genes such as *HES* (hairy and enhancer of split) and *HEY* (hairy/enhancer-of-split related with YRPW motif protein) genes.

Notch signaling plays important roles in the cornea development and homeostasis [9,10,11,12,13,14,15]. Hes1 has been shown to regulate the corneal development and the homeostatic function of LSCs in mice [12]. Moreover, Notch inhibition using a γ-secretase inhibitor in the human limbal epithelial cells in vitro reduced the amount of proliferating cells and increased keratin 3 (K3) expression; the opposite effect was observed after Notch activation with a non-immobilized recombinant Jagged1 (Jag1) protein [9]. A mechanism for corneal epithelial differentiation where Notch signaling is, in part, regulated by microRNA 31 (miR-31)/hypoxia-inducible factor 1 (FIH-1) has been proposed [16].

The function and regulation of Notch signaling in the human LSCs and corneal epithelial cells is largely unknown. With the aim to better understand the regulation of the human LSC differentiation and stratification, we investigated the role of Jag1-mediated Notch signaling activation in the cultivated human limbal epithelial cells (LECs).

## 2. Materials and Methods

### 2.1. Human Sclerocorneal Tissue

Human sclerocorneal tissue from 20- to 70-year-old healthy donors was obtained from different eye banks. Experimentation on human tissue adhered to the tenets of the Declaration of Helsinki. The experimental protocol was evaluated and exempted by the University of California Los Angeles Institutional Review Board (IRB#12-000363). The death-to-preservation time was less than 12 h, and the death-to-experiment time was less than 7 days.

### 2.2. Isolation and Cultivation of Limbal Epithelial Cells

LECs were isolated as previously described [17,18]. In brief, central 8-mm cornea was trephined, and residual iris, endothelium, Tenon’s capsule and conjunctiva were removed. To isolate LEC sheets or clusters, the rim was incubated with 2.4 U/mL dispase II (Roche, Indianapolis, IN, USA) at 37 °C for 2 h in DMEM/F-12 (Thermo Fisher Scientific, Waltham, MA, USA), followed by gentle scrapping. To isolate single LECs, cell sheets were further digested with 0.25% trypsin-1 mM EDTA (Thermo Fisher Scientific) for 5 min.

LSC cultures were performed as previously described [17]. Single LECs (used as a control) were seeded at a density of 300 cells/cm^2^ on sub-confluent 3T3-J2 mouse fibroblasts (3T3; Kerafast, Boston, MA, USA; Cat # EF3003, RRID: CVCL_W667) that had been growth-arrested with 4 µg/mL of mitomycin C (Sigma-Aldrich, St. Louis, MO, USA) for 2 h at 37 °C. Full thickness limbal explants (~2 mm^2^) were also obtained from the limbal region of the sclerocorneal rims. Explant tissues were cultured with the epithelial side facing up on either 12-well plastic culture plates, Tisseel fibrin glue (Baxter, Deerfield, IL, USA) or Polyethylene Terephthalate (PET) cell culture inserts of 1 µm pore size (Millipore, Burlington, MA, USA). One explant was used per well/cell culture insert. LSCs were cultured using supplemental hormone epithelial medium (SHEM) that consisted of DMEM/F12 medium supplemented with 5% fetal bovine serum (FBS; Thermo Fisher Scientific), N2 supplement (Thermo Fisher Scientific), 2 ng/mL of epidermal growth factor (EGF; Thermo Fisher Scientific), 8.4 ng/mL of cholera toxin (Sigma-Aldrich), 0.5 µg/mL of hydrocortisone (Sigma-Aldrich), 0.5% of dimethyl sulfoxide (DMSO; Sigma-Aldrich), penicillin-streptomycin (Thermo Fisher Scientific) and gentamicin/amphotericin B (Thermo Fisher Scientific). The culture medium was changed every 2-3 days.

Images of cell cultures were taken with an inverted DMIL LED microscope (Leica Microsystems, Wetzlar, Germany). Cell size was measured on 10× images by using Image J 1.50i software (US National Institutes of Health, Bethesda, MD, USA, RRID: SCR_003070). The percentage of cells ≤ 12 µm was calculated for each culture condition. Cell viability was determined by using the trypan blue (MP Bio, Irvine, CA, USA) dye exclusion test to quantify live and dead cells.

### 2.3. Notch Signaling Activation in the Limbal Epithelial Cells

To investigate the effect of Notch signaling activation on the LECs, a recombinant protein for rat Jag1 that is fused with the Fc region was used (R&D Systems, Minneapolis, MN, USA; Cat # 599-JG, RRID: Q63722.2) so that the Jag1 could be coated on the culture surface, and hence, immobilized to the substrate. First, either the tissue culture plate, fibrin or PET membrane were coated with anti-human IgG (Jackson ImmunoResearch, West Grove, PA, USA) for 30 min at 37 °C followed by 4 washes in 1X Phosphate-Buffered Saline (PBS; Thermo Fisher Scientific). Blocking was performed in 2% bovine serum albumin (BSA; Sigma-Aldrich) for 1 h at 37 °C. Incubation with the Jag1 (5 µg/mL) was done in 2% BSA for 2 h at 37 °C. Control cultures were set up in parallel and directly cultured on the uncoated surface.

Notch activation in the LECs was investigated by seeding LEC clusters on the Jag1-coated surface. LECs were allowed to attach and Notch activation was analyzed after 24 h by looking at the expression and the localization of the Notch 1 intracellular domain (N1IC). LN rat cells expressing N1 ectopically (LN1-7 cells) provided by Dr. Geraldine Weinmaster were used as a positive control [19]. LN1-7 cells were cultured in DMEM (Thermo Fisher Scientific) supplemented with 10% FBS. Notch activation experiments in the LECs after 24 h in the presence of the Jag1 recombinant protein were performed by using 3 different human sclerocorneal donor tissues. A minimum of 256 cells were analyzed per condition (*n* = 932 cells, control; *n* = 1086 cells, +Jag1 on plastic; *n* = 306 cells, +Jag1 on PET membrane; *n* = 256 cells, +Jag1 on fibrin). A minimum of 236 cells were analyzed for the LN1-7 control cell line (*n* = 352 cells, control; *n* = 345 cells, +Jag1 on plastic; *n* = 313 cells, +Jag1 on PET membrane; n = 236 cells, +Jag1 on fibrin). In addition to Jag1, DAPT (Sigma-Aldrich), the gamma-secretase Notch inhibitor, was added to the culture medium of the LECs at 20 µM for 24 h to assess Notch activation by looking at the expression and localization of N1IC. Two experiments from 2 different human sclerocorneal tissues were performed in the presence of Jag1 and DAPT. A minimum of 266 cells were analyzed per condition (*n* = 427 cells, control; *n* = 266 cells, +Jag1 on plastic; *n* = 353 cells, +Jag1+DAPT).

Limbal explants were placed in the middle of the Jag1-coated surface and allowed to grow until 80% confluence up to 10 days. LEC cultivation experiments in the presence and absence of the Jag1 recombinant protein were performed by using primary LECs in the form of limbal explants from 4 different human sclerocorneal donor tissues. The potential donor tissue differences were controlled by including a control group in the absence of Jag1 using each donor tissue on all the surfaces analyzed. Duplicates of the same condition were included in each of the experiments. Additionally, LECs in the form of single cells cultivated on 3T3 cells with SHEM were used as another control; this last experimental group was used to control for the quality of the sclerocorneal tissue by showing the growth of clonogenic LSCs.

### 2.4. Mitotic Arrest of the Stratified Limbal Epithelial Cultures and Analysis of Divisions

LEC cultures in the presence or absence (control) of Jag1 were growth arrested before cytokinesis completion to study the type of divisions at the basal layer of the limbal epithelium. At this stage, the nuclear envelope reforms but cells still share the cytoplasm. The aim was to study the proportion of asymmetric versus symmetric divisions in the stratified epithelium, and determine whether differences in the stratification are due to changes in the divisions as a consequence of Notch activation. For mitotic synchronization, nocodazole [20] (Sigma-Aldrich Cat # M1404, CAS ID: 31430-18-9) at 10 µM was added to the culture for 18 h, followed by a 3-h incubation with pyrimidyn-7 [21] (Abcam, Cambridge, MA, USA; Cat # ab144501, CAS ID: 1440126-94-2) for cytokinesis analysis. Two different human sclerocorneal donor tissues were used in these experiments.

Identification and orientation of arrested mitotic divisions was done by staining F-actin with Alexa Fluor 488 Phalloidin (Thermo Fisher Scientific) and pericentrin, a centrosome protein that serves as a multifunctional scaffold for anchoring numerous proteins. The expression pattern of the polarity protein partitioning defective protein 3 (Par3), involved in establishing apical-basal polarity and the alignment of the mitotic spindle with the polarity axis, was also studied in the mitotic divisions. Peripheral areas of the cultures within approximately 10 mm of the edge of the explant biopsy were examined for both control and Jag1 samples. To quantify the type of division (symmetric or asymmetric) at the basal layer, a total of 102–132 cells were analyzed.

### 2.5. Immunohistochemistry

Immunostaining was performed on cytospin slides, whole mounts and cryosections from cell culture sections. Cytospin slides were prepared by using the cytocentrifuged (Cytofuge; Thermo Fisher Scientific) and stored at -20 °C. Whole mounts were immunostained right after the culture period. Tissue sections of 8 µm thickness were obtained by using the CM3050 S Cryostat (Leica). Fixation was done by using 4% paraformaldehyde (PFA; Thermo Fisher Scientific) followed by blocking and permeabilization in PBS containing 1% bovine serum albumin (Sigma-Aldrich) and 0.5% Triton X-100 (Sigma-Aldrich) for 30 min. Incubation with primary antibodies (Table S1) was done overnight at 4 °C followed by incubation with secondary antibodies for 1 h at room temperature. Nuclei were counterstained with Hoechst 33342 at 4 µg/mL (Thermo Fisher Scientific). Images were acquired by using a Zeiss Image.A2 fluorescent microscope (Carl Zeiss Inc., Oberkochen, Germany) or a confocal microscope (Confocal Laser Scanning Microscopy, Olympus, San Jose, CA, USA).

Quantification of cells expressing high levels of p63α (p63α^bright^ cells) was done by using the Definiens Tissue studio^®^ 3 software (Definiens, Larchmont, NY, USA; RRID: SCR_014283) as previously reported [22] by quantifying 227-1175 cells per condition from 3 donor tissues depending on cell availability. Quantification of K14, K12, N1IC and Ki67 was done by using Image J software by counting 60–610 cells per condition from 3 donor tissues depending on cell availability. Quantification of the markers’ expression was performed after cell dissociation to better examine the expression in individual cells.

### 2.6. Quantitative RT-PCR (qRT-PCR)

Total RNA was extracted by using RNeasy Mini and Micro Kit (Qiagen, Hilden, Germany) and underwent DNase treatment (Ambion Inc., Austin, TX, USA). The quantity and quality of total RNA were assessed using a spectrophotometer (NanoDrop 1000; Thermo Fisher Scientific). Reverse-transcription was performed using the Superscript II RNase H2 reverse transcriptase kit (Thermo Fisher Scientific). The relative abundance of transcripts was detected by quantitative (q) RT-PCR (KAPA SYBR FAST qPCR Master Mix, Stratagene, San Diego, CA, USA). The housekeeping gene glyceraldehyde-3-phosphate dehydrogenase (GAPDH) was used to normalize the fluorescence level. The primers used for qRT-PCR are shown in the Table S2.

### 2.7. Air-lifting Induction

Air-lifting was induced in the LEC cultures from 3 different human sclerocorneal donor tissues by lowering the amount of culture medium to expose the cells to the air-liquid interface for 14 days. Air-lifted LEC cultures were then fixed in 4% PFA, embedded in optimal cutting temperature solution (Thermo Fisher Scientific) and stored at -80 °C. Hematoxylin and Eosin staining was performed to investigate the structure of the stratified cultures. Briefly, sections were placed in 0.1% Mayer’s Hematoxylin (Sigma-Aldrich) for 10 min, washed in running water for 5 min, counterstained in 0.5% Eosin Y solution (Sigma-Aldrich) for 15 s, dehydrated in increasing concentrations of ethanol, dipped in xylene (Sigma-Aldrich) for 5–6 times and mounted with Cytoseal XYL (Thermo Fisher Scientific).

Image J software was used to quantify (a) the amount of stratification (number of layers) in the different culture conditions given by the expression of K14, (b) the cell differentiation level by measuring the expression of K12, and (c) the number of basal cells per 100 µm on the cross-sections of the cultures. Three to 6 images of LEC culture cross-sections per donor tissue were examined to determine the number of layers, K12 expression and number of basal cells.

### 2.8. Quantification and Statistical Analysis

Data were statistically analyzed by using the Student’s *t*-test from at least three independent experiments. Error bars are expressed as the mean ± standard error of the mean (SEM). Significance was determined at *p* values < 0.05. Statistical details of experiments can be found in the figure legends.

## 3. Results

### 3.1. Jag1 is Expressed in the Limbal Epithelium and Activates Notch Signaling in LECs

**Figure 1:**
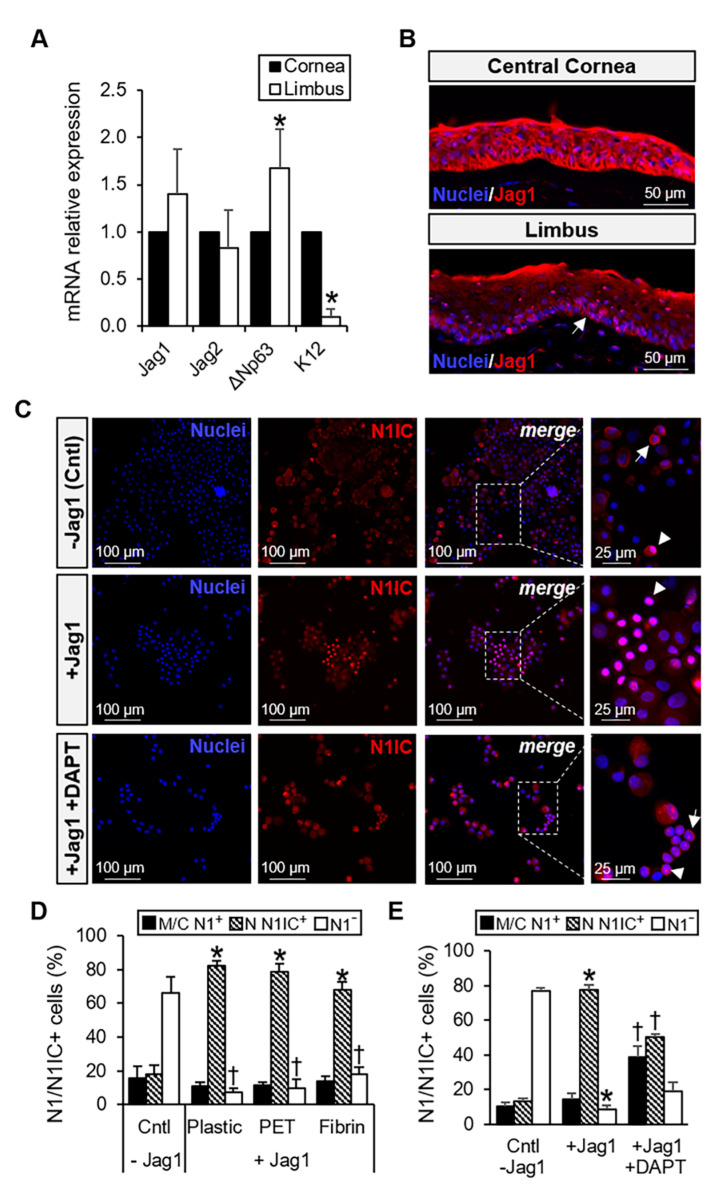
Jag1 is expressed in the limbal epithelium and activates Notch signaling in LECs after 24 h. (**A**) Relative mRNA expression of Jag1, Jag2, ΔNp63 and K12 in the human limbal and corneal epithelium. Jag1 mRNA expression level was similar in the limbus and central cornea. (**B**) At the protein level, Jag1 expression in human central cornea was homogeneous throughout the layers; Jag1 expression was mostly localized at the basal and immediate suprabasal layers of the limbal epithelium (arrow), as well as in the stromal cells. (**C**) Expression of N1IC in the nucleus of LECs is increased in the presence of Jag1 and decreased in the presence of Jag1 plus DAPT. The arrow indicates membrane/cytoplasmic expression of N1. The arrowhead indicates nuclear expression of N1IC. (**D)** Quantification of N1 shows a significant increase of nuclear N1IC in the Jag1 cultivated LECs on plastic, PET and fibrin substrates. In panel D, * indicates significant differences in the amount of N N1IC^+^ cells compared to control –Jag1. † indicates significant differences in the amount of N1^-^ cells compared to control –Jag1. (E) A significant decrease of nuclear N1IC is observed in the Jag1 plus DAPT group when compared to that in the Jag1 group. In panel E, * indicates significant differences in the amount of N N1IC^+^ and N1^−^ cells between the Jag1 group and the control group without Jag1. † indicates significant differences in the amount of N N1IC^+^ and M/C N1^+^ cells between the Jag1 plus DAPT group and Jag1 group. Data are represented as mean ± SEM. Abbreviations: Cntl: Control; C: cytoplasm; Jag1: Jagged 1; M: membrane; N: nucleus; N1: Notch 1; N1IC: Notch 1 intracellular domain; PET: Polyethylene Terephthalate. See also Figure S1.

We first investigated the expression levels of Jag1 in the human corneal and limbal epithelium. Jag1 mRNA expression level was comparable in both corneal and limbal epithelium (Figure 1A). Jag2 showed a decreased expression level compared to Jag1, although no statistically significant differences were found (Figure 1A). ΔNp63 and K12 were used as controls to determine whether isolation of epithelial cells from central cornea and limbus was successful (Figure 1A). At the protein level, Jag1 expression was observed throughout all epithelial layers in the central cornea and the expression pattern was homogeneous (Figure 1B); however, Jag1 was mostly localized at the basal and the immediate suprabasal layers of the limbal epithelium, as well as in the stromal cells.

The activation of Notch signaling was first confirmed in the freshly isolated primary LECs after 24 h of incubation with the recombinant protein Jag1 by analyzing the expression and localization of Notch 1 (N1) at the protein level. LN1-7 cells expressing N1 ectopically were used as the positive control [19]. A significantly high expression (> 70%; *p* ≤ 0.001) and nuclear localization of the N1 intracellular domain (N1IC) was found when LN1-7 cells were incubated with the immobilized Jag1 on all three types of surfaces (plastic, PET membrane and fibrin) compared to the uncoated control (Figure S1A,B). Morphology of LN1-7 cells was more elongated in the presence of Jag1 (Figure S1C).

When LECs were seeded with Jag1, we observed a significantly higher N1IC nuclear localization on plastic (86.2% ± 3.2%, *p* ≤ 0.0001), PET membrane (78.6% ± 5.1%, *p* = 0.004) and fibrin (68.2% ± 4.3%, *p* = 0.007) compared to that in the uncoated control (17.8% ± 5.5%) at 24 h (Figure 1C,D). No significant differences were found in the percentage of cells expressing N1 either on the membrane or the cytoplasm (range, 10.7% to 15.7%, *p* > 0.05; Figure 1D). These findings show that Jag1 could activate Notch signaling in the LECs. Notch signaling activation on fibrin was the weakest followed by PET membrane given by the percentage of nuclear N1IC^+^ cells (Figure 1D). A similar pattern was observed in the LN1-7 control cells (Figure S1B). When LECs were cultured on Jag1 and with the addition of DAPT in the medium, a significant decrease in the percentage of N1IC nuclear localization (50% ± 1.6%, *p* = 0.03) was observed compared to that in the LECs cultured on Jag1 without DAPT (77.4% ± 2.9%) at 24 h (Figure 1C,E). A significant increase of the cells expressing N1 on the membrane or cytoplasm was also observed in the LECs cultured on Jag1 and in the presence of DAPT (38.9% ± 5.9% versus 14.4% ± 3.3% in the Jag1 control, *p* = 0.04; Figure 1E). These findings suggest that DAPT was able to partially revert the effect of Jag1-induced Notch activation.

### 3.2. Jag1 Promoted Differentiation of LECs *In Vitro*

**Figure 2:**
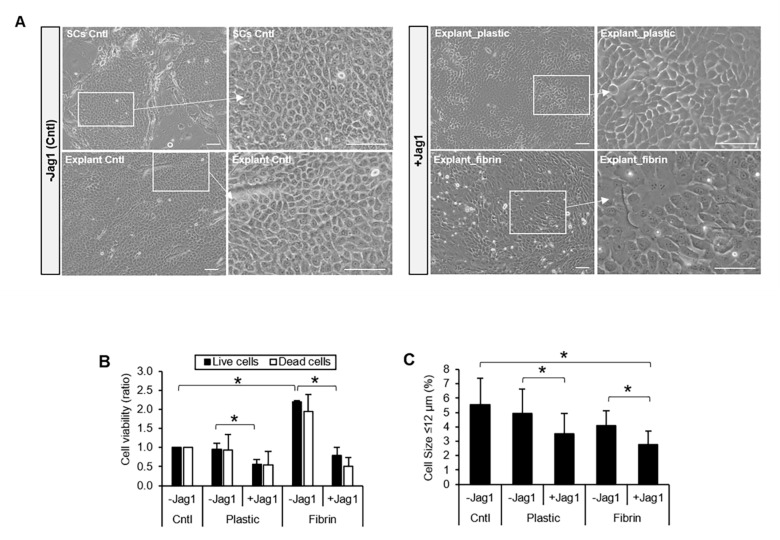

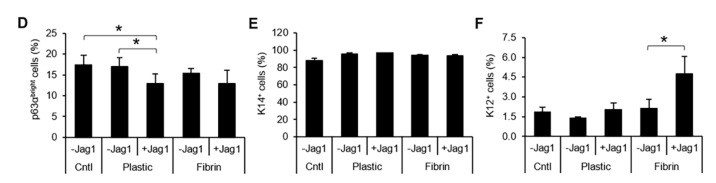
Jag1 promotes differentiation of LECs in vitro. (**A**). LECs cultivated with Jag1 in the presence and absence of fibrin gel showed a large, squamous-like cell morphology with less obvious cell-cell junctions. Scale bar: 50 µm. (**B**). Cell growth on plastic and fibrin was decreased in LECs cultivated with Jag1; cell viability in the Jag1 group was comparable to that of the control. (**C**). In the presence of Jag1, the percentage of small cells (≤ 12 µm) was decreased on plastic and fibrin. (**D**). The percentage of p63α^bright^ cells was decreased in the presence of Jag1; only LECs cultivated on plastic showed significant differences. (**E**). K14 expression was maintained in the LECs with Jag1. (**F**). The percentage of K12^+^ cells was increased in the presence of Jag1; only LECs cultivated on fibrin showed significant differences. -Jag1 Cntl cultures correspond to single LSCs growing on 3T3 cells. Plastic and Fibrin (-/+Jag1) correspond to explant cultures. In panels **B**, **C**, **D** and **F**, * indicates significant differences between the groups marked by the key. Data are represented as mean ± SEM. Abbreviations: Cntl: Control; SCs: single cells; K12: cytokeratin 12; K14: cytokeratin 14; Jag1: Jagged 1. See also Figure S2.

We next investigated whether Notch signaling activation by Jag1 had any effect on the LEC phenotype in vitro. For this purpose, limbal explant cultures were used. LECs in the control cultures showed a small, compact and cuboidal cell morphology with defined cell-cell junctions (Figure 2A, -Jag1 Cntl). LECs cultured with Jag1 showed a large, squamous-like cell morphology with less obvious cell-cell junctions (Figure 2A, +Jag1). This phenotype in the cells with Jag1 suggested a more differentiated cell morphology compared to that in the control culture.

Cell viability analysis showed a reduction in the number of live cells in the limbal explant cultures in the presence of Jag1, both in the presence (2.8-fold lower, *p* = 0.009; Figure 2B) and absence of fibrin (1.7-fold lower, *p* = 0.04; Figure 2B) compared to their respective controls. The number of dead cells in the presence of Jag1 was comparable to the control without Jag1 (*p* > 0.05), indicating that the reduction in the number of cells in the presence of Jag1 is not due to an increase in cell death.

We next investigated the LSC population in the cultures by analyzing the percentage of cells with a small cell size (≤ 12 µm), a high expression level of p63α [23] and expression of K14. The percentage of small cells (≤ 12 µm) decreased when LSCs were cultured in the presence of Jag1 on plastic (1.4-fold reduction, p = 0.03) and fibrin (1.5-fold reduction, p = 0.01) compared to the limbal explant control cultures (Figure 2C). The percentage of p63α high-expressing cells (p63α^bright^ cells) decreased in the presence of Jag1 but the difference reached statistical significance when LECs were cultured on plastic (12.8% ± 2.3% vs. 17% ± 2.2% in the explant control cultures, p = 0.02; Figure 2D and Figure S2D). The percentage of K14^+^ cells did not change in the presence of Jag1 (p > 0.05; Figure 2E and Figure S2D). The percentage of terminally differentiated K12^+^ cells increased in the presence of Jag1 and the difference reached statistical significance when LECs were cultured on fibrin (4.7% ± 1.4% vs. 2.1% ± 0.6% in the control, p = 0.04; Figure 2F and Figure S2D). Altogether, these results suggest a decrease in the LSC population and an increase in the differentiated K12^+^ cell population in the presence of Jag1.

### 3.3. Jag1-Mediated Notch Activation Reduced Stratification and Promoted Differentiation of LECs upon Air-Lifting

Air-lifting is a common maneuver to induce epithelial stratification and differentiation mimicking the in vivo process that was first described in mouse [24] and human [25] skin keratinocytes. Air-lifting is induced to produce cell sheets of cultured LECs that are equivalent to the in vivo limbal epithelium. After the limbal explant cultures reached approximately 80% confluence, the differences in the stratification and differentiation induced by air-lifting were analyzed.

**Figure 3:**
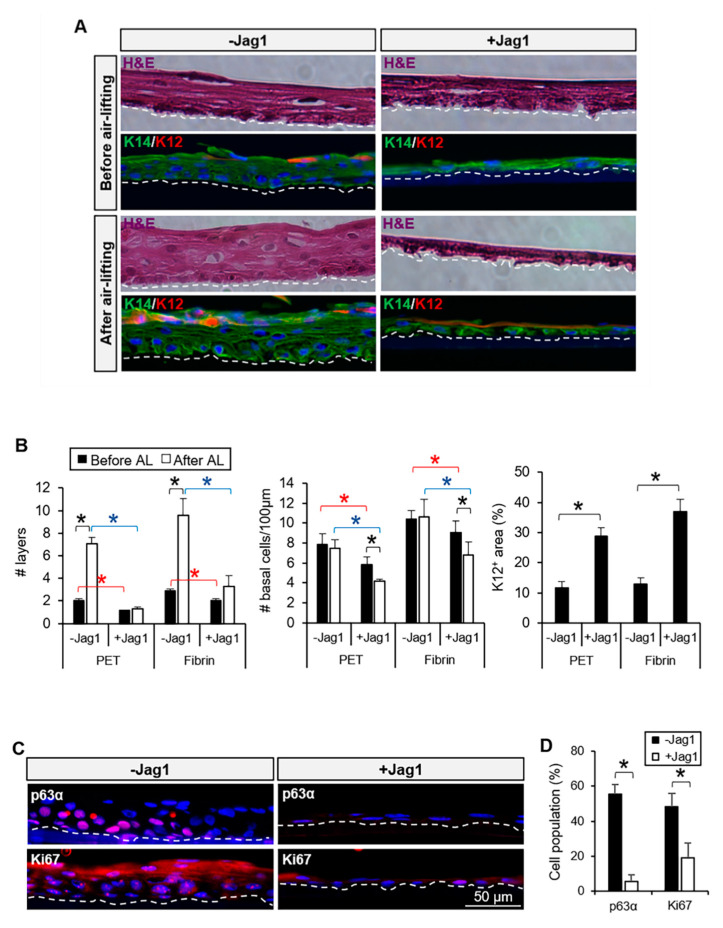
Jag1-mediated Notch activation reduced stratification and promoted differentiation of LECs. (**A**). Stratification of the cultivated LECs in the presence of Jag1 was reduced before and after air-lifting induction. Differentiation was maintained after air-lifting in the Jag1 group at the superficial layer(s). (**B**). The number of layers and number of cells per µm at the basal layer were reduced in the Jag1 cultures; the K12^+^ area in the presence of Jag1 was increased compared to the control. (**C**). Expression of p63α and Ki67 was reduced at the basal layer of the cultivated LECs with Jag1. (**D**). Quantification of the percentage of cells positive for p63α and Ki67 showed a significant reduction in the cultivated LECs with Jag1. The dotted line in A and C panels delineates the BM. In panels B and D, * indicates significant differences between the groups marked by the key. Data are represented as mean ± SEM. Abbreviations: BM: basement membrane; H&E: hematoxylin and eosin; Jag1: Jagged 1; K12: cytokeratin 12; K14: cytokeratin 14; PET: Polyethylene Terephthalate.

Before air-lifting, we observed that cultures in the presence of Jag1 were less stratified, and contained a lower number of cell layers on both PET membrane (1.1 ± 0.2 vs. 2.0 ± 0.2 layers in the control, *p* = 0.001; Figure 3A,B) and fibrin (2.0 ± 0.1 vs. 2.9 ± 0.1 layers, *p* = 0.001; Figure 3A,B). The number of basal cells per 100 µm on the cross-sections was also decreased in the presence of Jag1 on both PET (*p* = 0.005; Figure 3A,B) and fibrin (*p* = 0.02; Figure 3A,B). Basal cells in the presence of Jag1 were more elongated than the cells in the control (Figure 3A). After air-lifting, the difference in the stratification further increased. Cultures in the presence of Jag1 were less stratified and contained a lower number of cell layers on both PET membrane (1.3 ± 0.1 vs. 7.1 ± 0.6 layers in the control, *p* = 0.00003; Figure 3A,B) and fibrin (3.3 ± 0.9 vs. 9.6 ± 1.5 layers, *p* = 0.0001; Figure 3A,B). The number of basal cells per 100 µm after air-lifting was also decreased in the presence of Jag1 on both PET (*p* = 0.02; Figure 3A,B) and fibrin (*p* = 0.0004; Figure 3A,B). Basal LECs with Jag1 after air-lifting were more flat, and the number of basal cells per 100 µm decreased compared to the control cultures and with Jag1 before air-lifting induction (*p* = 0.01; Figure 3A,B). After air-lifting, the amount of K12^+^ area in the presence of Jag1 increased compared to the control on PET (28.8% ± 2.9% vs. 11.7% ± 2.0% in the control, *p* = 0.0003) and fibrin (36.9% ± 4.0% vs. 13.0% ± 2.1%, *p* ≤ 0.001; Figure 3A,B). 

To further characterize the basal cells, we quantified the expression of the proliferation markers p63α and Ki67. P63α is mostly expressed at the basal and immediate suprabasal layers of the human limbus (Figure S2A). In the presence of Jag1, the expression of both p63α and Ki67 was drastically reduced (all p ≤ 0.01) compared to that in the control (Figure 3C,D).

### 3.4. Jag1 Decreased Asymmetric Divisions in Basal LECs

**Figure 4:**
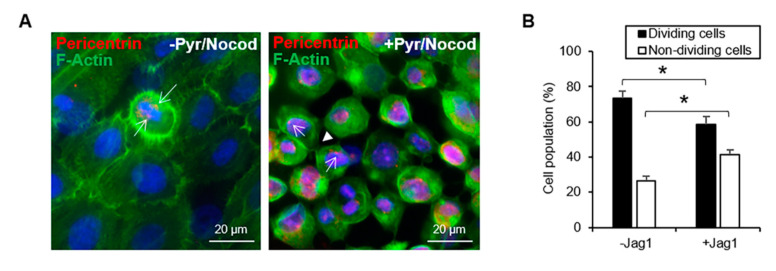

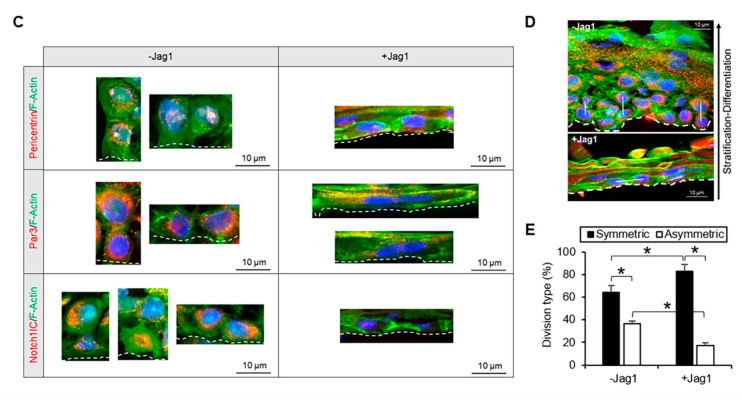
Jag1 Decreased Asymmetric Divisions in basal LECs. (**A**). Pericentrin stained both poles of the mitotic spindle in cells undergoing mitosis before and after a combined treatment of nocodazole and pyrimydine-7. Arrows indicate pericentrin staining. Arrowhead indicates cleavage furrow and contractile ring stained with F-actin. (**B**). The percentage of dividing cells was significantly reduced in the presence of Jag1. (**C**). Both in the presence and absence of Jag1, pericentrin was identified in the daughter cells of symmetric and asymmetric divisions, and together with F-actin helped identify the orientation of the mitotic spindle. In control cultures without Jag1, Par3 was expressed at the apical-lateral membrane of cells; in the Jag1 cultures, the expression of Jag1 was more delocalized and scattered in the cells. In control cultures, N1IC was expressed in the nucleus of basal cells dividing asymmetrically; cells dividing symmetrically had mostly cytoplasmic expression of N1IC; in Jag1 cultures, cells dividing symmetrically expressed nuclear N1IC. The basement membrane is delineated by the dotted line. (**D**). The plane of asymmetric and symmetric divisions (represented by the white line) is shown in the presence and absence of Jag1 in cross-sections of cultivated LECs. A decrease in the number of asymmetric divisions was observed in LECs cultivated with Jag1. The basement membrane is delineated by the dotted line. (**E**). The percentage of asymmetric divisions was reduced in the presence of Jag1. In panels **B** and **E**, * indicates significant differences between the groups marked by the key. Data are represented as mean ± SEM. Abbreviations: Jag1: Jagged 1; Nocod: Nocodazole; Notch1IC: Notch 1 intracellular domain. Par3: Partitioning defective protein 3; Pyr: Pyrimidyn-7. See also Figure S2.

After treatment with nocodazole [20] and pyrimidyn-7 [21], the mitotic divisions were arrested before cytokinesis completion in the cultured LECs (Figure 4). In non-arrested LECs, pericentrin was observed in both poles of the mitotic spindle of the cells that were undergoing mitosis (Figure 4A, left), confirming the specificity of the pericentrin antibody in the LECs. Pericentrin was also present in arrested cell cultures before cytokinesis (Figure 4A, right). Pericentrin identifies the orientation of the mitotic spindle by labeling the centrosome of the resulting daughter cells during the division [26]. In the arrested LECs cultures, F-actin and pericentrin allowed the identification of the daughter cells, still connected by the cytoplasm, and the orientation of the mitotic spindle in the dividing cells. Dividing cells were identified by the presence of the cleavage furrow and contractile ring after mitotic arrest. In culture cross-sections, we observed that 73.5% of the cells were dividing in the control cultures (Figure 4B). However, in the presence of Jag1, there was a significant reduction of the dividing cells (58.7%, *p* = 0.04; Figure 4B).

The expression pattern of Par3 (Partitioning defective protein 3) was analyzed to determine how polarity was established during cell division in the presence and absence of Jag1. In asymmetric divisions, the mitotic spindle is perpendicular to the basement membrane (BM), generating two daughter cells with different potential, one that remains undifferentiated and in contact with the BM, and another cell more differentiated cell in the suprabasal layer (Figure 4D). Par3 has a crescent-like expression pattern in asymmetric divisions and it is mostly localized at the apical-lateral membrane of the suprabasal cell [27,28]. In symmetric divisions, the mitotic spindle is parallel to the BM, generating two daughter cells with the same potential that remain in contact with the BM (Figure 4D). Par3 expression in symmetric divisions is equally distributed in the two daughter cells, and preferentially located at the apical-lateral membrane of the cells [27,28].

By analyzing pericentrin and F-actin staining, in control cultures, 65.5% of divisions were symmetric and 34.5% were asymmetric (Figure 4C,E). In contrast, in the presence of Jag1, asymmetric divisions were decreased to 17% (*p* = 0.04) and symmetric divisions were increased to 83% (*p* = 0.04; Figure 4C,E). In the control, Par3 had a crescent-like pattern expression, mostly localized in apical-lateral membrane of the suprabasal cell resulting from an asymmetric division (Figure 4C); Par3 was equally distributed in the apical lateral membrane of the basal daughter cells resulting from a symmetric division (Figure 4C). In the presence of Jag1, Par3 expression pattern was more scattered and disperse within the cytoplasm of the basal cells compared to that in the controls (Figure 4C). Basal cell polarity in the presence of Jag1 did not seem as pronounced compared to the control cultures. The specificity of the Par3 antibody in the limbal epithelial cells was confirmed on sections of human sclerocorneal tissue (Figure S2B). Expression of N1IC was also analyzed to determine where Notch signaling was activated in the mitotic divisions. N1IC expression in the adult human limbus was mostly detected at the suprabasal layers and sporadically in the basal cells (Figure S2C). In the control LECs without Jag1, N1IC expression was mostly localized in the cytoplasm during symmetric divisions (Figure 4C). In asymmetric divisions, nuclear N1IC was observed in the basal cells that are in contact with the BM (Figure 4C), and in the cytoplasm of the suprabasal cells. In the presence of Jag1, N1IC was detected both in the cytoplasm and nuclei of the two daughter cells during the mitotic division before complete cytokinesis (Figure 4C), suggesting Notch activation during cell division.

## 4. Discussion

Notch signaling is a central pathway that regulates cell fate decisions during development and differentiation in adult tissues, and it is well studied in the embryonic and adult epidermis. Notch-mediated cell differentiation and Jag1 expression pattern at the basal and immediate suprabasal layers of the epidermis has been previously reported [29,30]. Understanding the regulation of differentiation and stratification in the limbal epithelium is fundamental to develop new treatment strategies. The findings in the current study are consistent with those previously obtained by our group [31] and others [9,10,11,12,13] in that Notch signaling pathway is present in the limbal epithelium where LSCs are located.

Jag1 expression pattern in the human sclerocorneal limbal tissue suggests a functional role of this ligand in the LSC population *in vivo*. Notch signaling was robustly activated in the LECs by using the immobilized ligand Jag1 on different culture substrates. Although in a weaker manner, Notch signaling was activated in the LECs cultured on fibrin. This is possibly due to the degradation of fibrin and, hence, the loss of Jag1 during the culture period. DAPT was able to partially suppress Notch activation induced by Jag1 in the LECs. However, DAPT was not able to return the level of Notch signaling activation to the basal level observed in the control LECs without Jag1. These results suggest that the activation exerted by the Jag1 ligand is stronger than the effect of DAPT. A higher concentration of DAPT might be needed. Moreover, since DAPT is a broad-spectrum inhibitor, it might be blocking some other signaling pathways, and, hence, being less efficient at blocking Notch signaling. We have previously shown that more specific Notch small molecule inhibitors such as SAMH1 can be more efficient at inhibiting Notch signaling in the LECs at some concentrations [31]. Moreover, Jag1 in combination with either DAPT or SAHM1 decreased Notch signaling activation in the LN1-7 cells, given by the percentage and localization of the N1IC [31].

Jag1-mediated Notch activation decreased the stem cell population in the LEC cultures, as evidenced by a reduced amount of small cells and p63α^bright^ cells, and increased differentiation given by the increased number of mature K12^+^ cells. Differentiation induced by Jag1-mediated Notch has been previously observed in several other type of epithelia [32,33] and in the eye [34]. Contrary to our findings, a previous study showed an increase in human corneal epithelial proliferation and decreased differentiation upon Notch activation by Jag1 [9]. Differences between our study and Ma et al.’s study might be due to the level of Notch activation. In Ma et al.’s study, Jag1 was added to the culture medium and incubated with the LECs for 2 days, whereas in our study, Jag1 was immobilized on the surface. It has been reported that robust Notch signaling activation requires the stabilization/immobilization of the ligand [35,36]. Discrepancies in these findings are likely due to the method of Jag 1 activation.

Upon Jag1-mediated Notch activation, proliferation and stratification were reduced and differentiation was increased. P63 is required for the development of stratified epithelia [37,38] by regulating proliferation and differentiation of keratinocytes [39]. Moreover, it has been shown that Notch signaling represses p63 expression in the developing epidermis in mice [40]. In the developing mouse cornea, upregulation of p63 in the basal cells of the epithelium by activation of BMP4 promotes corneal epithelial stratification [41]. Our data is consistent with the inverse correlation between Notch signaling activation and proliferation in the corneal epithelium that was previously reported [11].

Moreover, our findings suggest that Notch signaling regulates symmetric and asymmetric divisions. The coordination of the mitotic spindle orientation with cell polarity is essential for asymmetric divisions at the basal layer [42]. In symmetrical cell divisions, the cellular components are equally distributed to the two daughter cells. By contrast, asymmetrical cell division involves unequal partitioning of the cellular components in the two daughter cells, generating in one of the cells with a more differentiated phenotype [43]. Levels of p63 control the mitotic spindle orientation. In particular, high p63 expression level promotes asymmetric divisions [38]. By the expression and localization of pericentrin (from the centrosome), F-actin (to observe the cleavage furrow and contractile ring) and Par3 (polarity protein at the apical membrane of polarized basal cells) [28], we were able to determine the orientation of the mitotic spindle and hence identify whether the division plane was parallel (symmetric division) or perpendicular to the BM (asymmetric division) in cultured LECs. In the current study, Notch signaling activation by Jag1 increased symmetrical divisions in the basal LECs where the expression level of p63 was lower compared to the control. Reduction of proliferation and asymmetric divisions at the basal layer likely reduced the stratification of the LECs. The role of asymmetric divisions in the induction of stratification and differentiation of the mammalian epidermis has been proposed before [44]. The level of Notch signaling is important to maintain the balance between epithelial cell proliferation and differentiation as previously shown in the mouse epidermis [44]. Moreover, miR-31 has been shown to downregulate FIH-1 and enhance stratified epithelial differentiation via Notch signaling [16].

Our study suggests that Notch signaling also regulates cell polarity in the LECs. In the presence of Jag1, the basal cell polarity was less prominent, given by the scattered distribution of Par3 within the cells. Changes in the Par3 expression pattern together with p63 downregulation might be responsible for the decreased asymmetric divisions at the basal layer [27,28]. Furthermore, the cells at the basal layer displayed a more differentiated morphology and phenotype, resembling that of the suprabasal epithelial cells in the limbus. Changes in the Par3 expression pattern have been proposed to cause initial expansion and later decline of stem cells, accompanied by an enrichment of committed progenitors and increased epithelial differentiation [45]. In addition, overexpression of Notch signaling promotes the dysregulation of Par3 generating a disperse Par3 pattern and reduction of asymmetric divisions [46]. Thus, the combination of a lower proliferation rate, decreased proportion of asymmetric divisions and the changes in Par3 expression pattern at the basal layer might be responsible for the reduction of stratification while promoting the differentiation of the basal LECs. 

In control cultures, N1IC was detected in the nucleus of the basal cells that are dividing asymmetrically and mostly in the cytoplasm of basal cells that are undergoing symmetrical divisions, possibly indicating that Notch signaling is active in basal cells dividing asymmetrically but not symmetrically. The asymmetric inheritance of Notch receptors and Notch effectors such as Numb has been proposed in mammalian neurons [47,48] and epidermis [49,50]. In contrast, in the presence of Jag1, N1IC was localized in the nucleus in both dividing cells in symmetric divisions. This aberrant Notch activation at the basal layer might promote symmetric divisions. The levels of Notch signaling activation are important for the differential regulation of quiescence, renewal and differentiation as previously shown [51,52].

**Figure 5:**
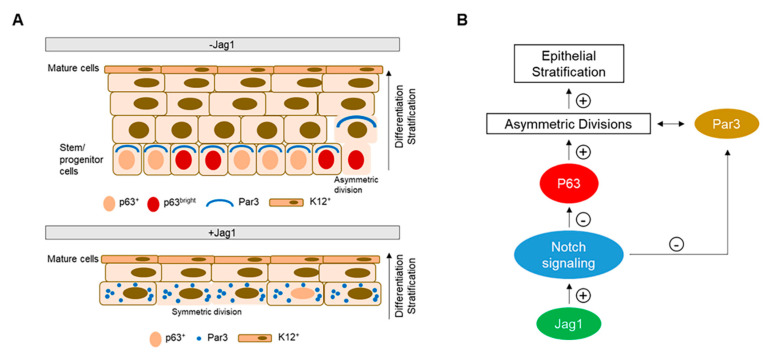
Proposed model of stratification-differentiation of the limbal epithelium. (**A**). In the absence of the recombinant protein for Jag1 (control), basal stem cells expressing p63 retain the capacity to divide asymmetrically generating two daughter cells, a new stem cell and a suprabasal more differentiated cell. The orientation of the mitotic spindle is controlled by the polarity proteins such as Par3 that are distributed on the apical-lateral membrane of polarized basal cells. Differentiated K12^+^ cells are present at the superficial layer(s). In the presence of Jag1, basal cells in direct contact with Jag1 have a scattered Par3 distribution, p63 expression is low, and there is a decrease in the proportion of asymmetric divisions. As a consequence, the stratification of the epithelium is reduced. Differentiated K12^+^ cells are still present at the superficial layer(s). (**B**). Schematic diagram showing that upon Jag1-mediated Notch activation, the expression of p63 is downregulated. P63 is the main driver of epithelial stratification. High levels of p63 promote asymmetric divisions, which in turns increases the stratification of the epithelium. Also, Notch signaling directly affects the expression of Par3. Overexpression of Notch signaling dysregulates Par3 expression and decreases asymmetric divisions.

Based on our observations, we propose a simplified model of stratification and differentiation of the human LECs in which low level of Notch signaling preserves the p63α^bright^ stem/progenitor cell population and maintains polarity to divide asymmetrically at the basal layer (Figure 5A,B). By contrast, upon Notch signaling activation by Jag1 in LECs, p63 is downregulated, proliferation is reduced, and polarity is lost. There is also a loss of the p63α^bright^ stem/progenitor cell population at the basal layer. As a result, asymmetric divisions are reduced followed by stratification. In addition, Notch activation by Jag1, promoted differentiation of LECs as evidenced by an increase of K12 expression (Figure 5A,B). These results highlight the importance of controlling the levels of Notch signaling in the in vitro cultivation of LECs. The reconstruction of a functional limbal epithelium ex vivo might require sporadic and controlled activation of Notch signaling to start differentiation without exhausting the stem cell population. Further studies are needed to confirm the proposed mechanism and the role of other signaling pathways in this model. The study of basic mechanisms in the limbal epithelium is fundamental to understand the differentiation program, and be able to modulate Notch signaling pathway in vitro to design future cell therapies.

## Supplementary Materials

The following are available online at https://www.mdpi.com/2073-4409/9/9/1945/s1, Table S1: Primary antibodies used for immunohistochemistry. Table S2: Primers used for qRT-PCR. Figure S1: Jag1-mediated Notch signaling activation in LN1-7 control cell line after 24 h. Figure S2: Immunostaining of the human limbus on tissue sclerocorneal sections and phenotypic characterization of the LEC cultivated in the presence/absence of Jag1.
