# Supplementary material for: Role of Jagged1-mediated Notch Signaling Activation in the Differentiation and Stratification of the Human Limbal Epithelium
Source: Cells. 2020 Aug 22;9(9):1945. doi: 10.3390/cells9091945 (PMC7564045; doi:10.3390/cells9091945)
Supplement: Supplementary file 1 [file cells-09-01945-s001.pdf]

Supplementary Materials

Table S1. Primary antibodies used for immunohistochemistry.

Proteins	Dilution	Source and Catalogue #
**K12**	1:100	Santa Cruz Biotechnology sc-25722
**K14**	1:50	Fisher Scientific MS-115-R7
**p63α**	1:100	Cell Signaling Technology #4892
**Cleaved Notch1**	1:100	Cell Signaling Technology #4147
**Jag1**	1:100	Abcam ab7771
**Pericentrin**	1:100	Covance #83351
**Par3**	1:100	R&D Systems
**Ki67**	1:200	DAKO M7240

Abbreviations: K12, Cytokeratin 12; K14: Cytokeratin 14; Jag1: Jagged1; Par3: Partitioning defective protein 3.

Table S2. Primers used for qRT-PCR.

Genes	Forward primer (5'-3')	Reverse primer (3'-5')
**GAPDH**	CGACCACTTTGTCAAGCTCA	AGGGGTCTACATGGCAACTG
**Jag1**	CCATTTCTGCTGAAGATATACGG	CACAGTTAAGACAGAGCTCAGCA
**Jag2**	TGGGACTGGGACAACGATAC	AGTGGCGCTGTAGTAGTTCTC
**ΔNp63**	TCCATGGATGATCTGGCAAGT	GCCCTTCCAGATCGCATGT
**K12**	CCAGGTGAGGTCAGCGTAGAA	CCTCCAGGTTGCTGATGAGC

Abbreviations: GAPDH: Glyceraldehyde 3-phosphate dehydrogenase; Jag1: Jagged 1; Jag2: Jagged 2; K12, Cytokeratin 12.

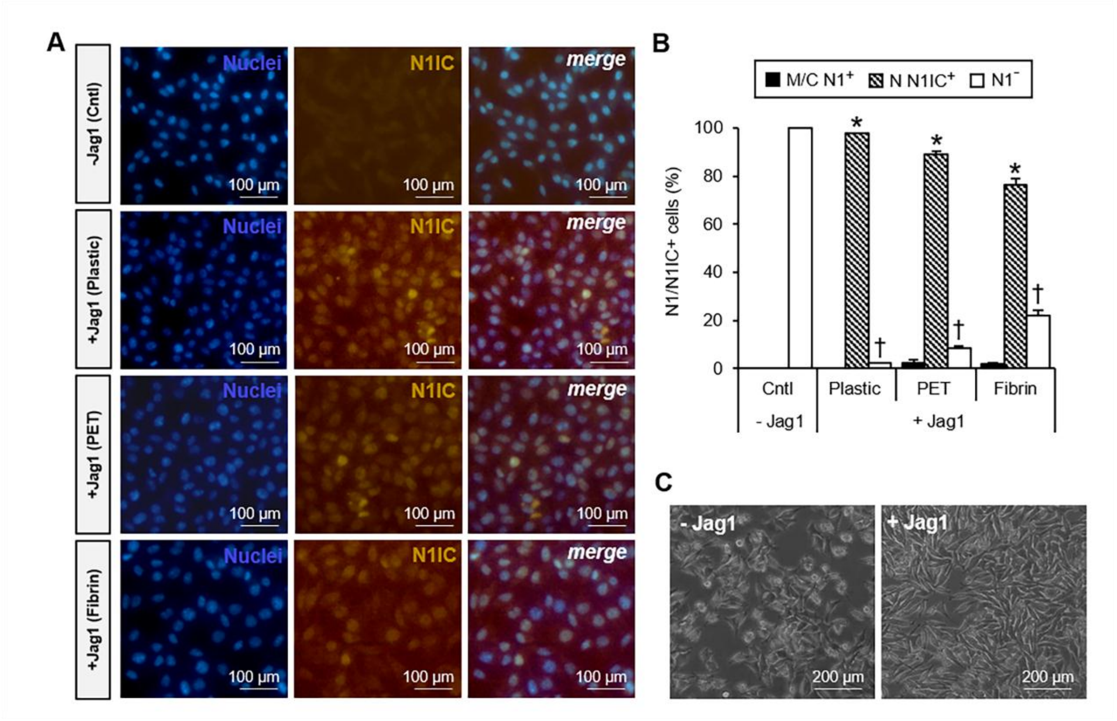

Figure S1. Jag1-mediated Notch signaling activation in LN1-7 control cell line after 24 hours. **A.** Expression of N1IC in the nucleus of LN1-7 cells increased in the presence of Jag1. **B.** Quantification of N1 shows a significant increase of nuclear N1IC in the Jag1 cultivated LN1-7 cells on plastic, PET and fibrin substrates. **C.** Cell morphology of LN1-7 cells in the presence of Jag1 was elongated and spindled-shape like. *Significant differences in the amount of N N1IC^{+} cells compared to control –Jag1. †Significant differences in the amount of N1^{-} cells compared to control –Jag1. Data are represented as mean ± SEM. Abbreviations: Cntl: Control; C: cytoplasm; Jag1: Jagged 1; M: membrane; N: nucleus; N1: Notch 1; N1IC: Notch 1 intracellular domain; Jag1: recombinant protein for Jag1; PET: Polyethylene Terephthalate.

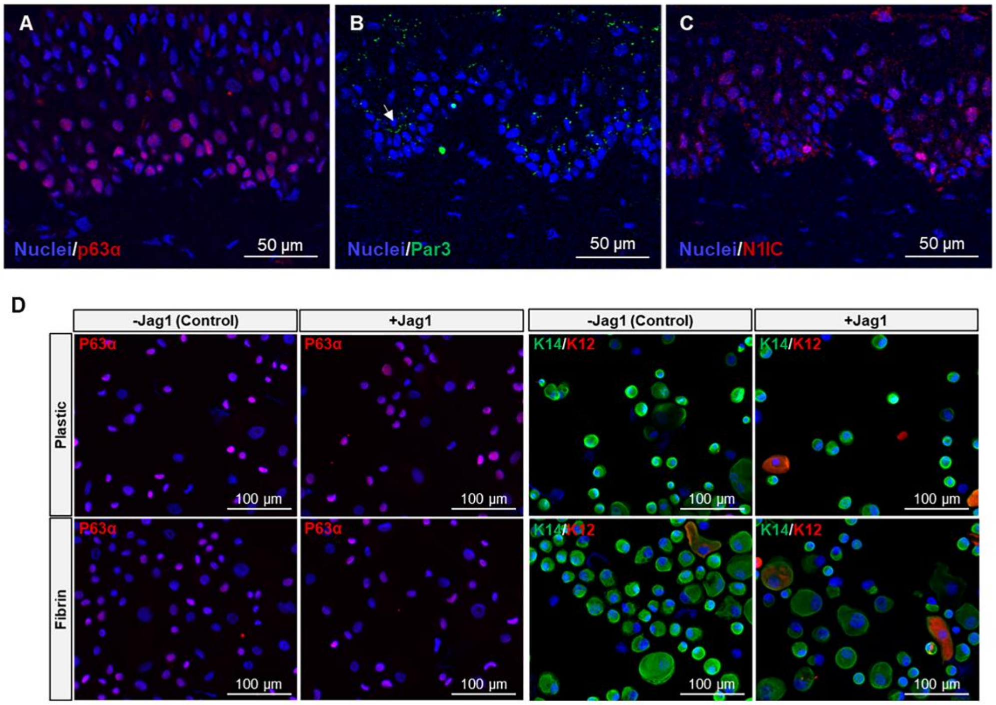

Figure S2. Immunostaining of the human limbus on tissue sclerocorneal sections and phenotypic characterization of the LEC cultivated in the presence/absence of Jag1. **A.** p63α predominantly expressed at the basal and suprabasal layers of the limbus. **B.** Par3 expression showing a crescent-like pattern at the basal layers (arrow) and a more scattered distribution suprabasally. **C.** N1IC predominantly expressed at the suprabasal layers and some cells at the basal cells. D. The percentage of p63α^{bright} cells was reduced in the presence of Jag1; the percentage of K12^{+} cells was increased in the presence of Jag1. Abbreviations: N1IC: Notch 1 intracellular domain; Par3: Partitioning defective protein 3.
